# High-Dose Statin Pretreatment Decreases Periprocedural Myocardial Infarction and Cardiovascular Events in Patients Undergoing Elective Percutaneous Coronary Intervention: A Meta-Analysis of Twenty-Four Randomized Controlled Trials

**DOI:** 10.1371/journal.pone.0113352

**Published:** 2014-12-04

**Authors:** Le Wang, Pingan Peng, Ou Zhang, Xiaohan Xu, Shiwei Yang, Yingxin Zhao, Yujie Zhou

**Affiliations:** Beijing Anzhen Hospital, Capital Medical University, Beijing Institute of Heart Lung and Blood Vessel Disease, The Key Laboratory of Remodeling-Related Cardiovascular Disease, Ministry of Education, Beijing 100029, China; Istituto Clinico S. Ambrogio, Italy

## Abstract

**Background:**

Evidence suggests that high-dose statin pretreatment may reduce the risk of periprocedural myocardial infarction (PMI) and major adverse cardiac events (MACE) for certain patients; however, previous analyses have not considered patients with a history of statin maintenance treatment. In this meta-analysis of randomized controlled trials (RCTs), we reevaluated the efficacy of short-term high-dose statin pretreatment to prevent PMI and MACE in an expanded set of patients undergoing elective percutaneous coronary intervention.

**Methods:**

We searched the PubMed/Medline database for RCTs that compared high-dose statin pretreatment with no statin or low-dose statin pretreatment as a prevention of PMI and MACE. We evaluated the incidence of PMI and MACE, including death, spontaneous myocardial infarction, and target vessel revascularization at the longest follow-up for each study for subgroups stratified by disease classification and prior low-dose statin treatment.

**Results:**

Twenty-four RCTs with a total of 5,526 patients were identified. High-dose statin pretreatment was associated with 59% relative reduction in PMI (odds ratio [OR]: 0.41; 95% confidence interval [CI]: 0.34–0.49; P<0.00001) and 39% relative reduction in MACE (OR: 0.61; 95% CI: 0.45–0.83; P = 0.002). The benefit of high-dose statin pretreatment on MACE was significant for statin-naive patients (OR: 0.69; 95% CI: 0.50–0.95; P = 0.02) and prior low dose statin-treated patients (OR: 0.28; 95% CI: 0.12–0.65; P = 0.003); and for patients with acute coronary syndrome (OR: 0.52; 95% CI: 0.34–0.79; P = 0.003), but not for patients with stable angina (OR: 0.71; 95% CI 0.45–1.10; P = 0.12). Long-term effects on survival were less obvious.

**Conclusions:**

High-dose statin pretreatment can result in a significant reduction in PMI and MACE for patients undergoing elective PCI. The positive effect of high-dose statin pretreatment on PMI and MACE is significant for statin-naïve patients and patients with prior treatment. The positive effect of high-dose statin pretreatment on MACE is significant for patients with acute coronary syndrome.

## Introduction

Percutaneous coronary intervention (PCI) is an important method in the treatment of coronary artery disease. Periprocedural myocardial infarction (PMI), characterized by cardiac biomarker elevation, is a common complication of PCI [1]. Studies have suggested that side branch occlusion, distal embolism, coronary dissection, disruption of collateral flow, and inflammation can result in PMI [1], [2]. Although most patients who have PMI remain asymptotic and have no change in cardiac function, PMI has been associated with higher mortality [3]. A meta-analysis showed that even small increases in creatine kinase-MB (CK-MB) are associated with significantly higher risk of death during follow-up [4]. Several therapeutic strategies, including statins, antithrombotic agents [5], [6], and β-blockers [7], [8], have been suggested to reduce PMI. Statins, which inhibit hydroxymethylglutaryl-CoA reductase, have been demonstrated to have pleiotropic effects, including rapid anti-inflammatory and antithrombotic properties [9], [10], antioxidant effects [11], improvement of endothelial dysfunction [12], and stabilization of atherosclerotic plaques [13]. Therefore, statins are regarded as an important agent for the prevention of PMI.

A 2011 meta-analysis [14] comprising 3341 patients from 13 randomized, controlled trials (RCTs) indicated that high-dose statin pretreatment is associated with a significant reduction in PMI and major adverse cardiac events (MACE) in patients undergoing PCI within thirty days. However, more recent clinical trials showed that early use of high-dose statin before PCI did not reduce the occurrence of PMI or improve the long-term clinical outcomes [15]–[18]. Another more recent meta-analysis of 14 RCTs with 3146 patients [19] showed that high-dose statin loading prior to PCI reduced clinical events for patients with acute coronary syndrome (ACS) but not stable angina. However, the latter study was not exhaustive and did not consider subgroups receiving chronic statin therapy prior to the high dose pretreatment. Therefore, a more comprehensive analysis of the benefits of high-dose statin reloading before PCI, including patients under long-term statin therapy, is needed. Thus, we performed a meta-analysis of 24 RCTs comprising 5,526 patients to reevaluate the efficacy of a short-term high-dose statin pretreatment to prevent PMI and MACE in patients undergoing elective PCI.

## Methods

### Search strategy and inclusion criteria

In this meta-analysis, two authors independently conducted a thorough search of the PubMed/Medline database for the reports of all RCTs conducted until January 2014 that compare the clinical outcomes of high-dose statin pretreatment with no-statin or low-dose statin pretreatment in patients undergoing elective PCI. Search keywords were “statins”, “statin”, “atorvastatin”, “rosuvastatin”, “cervastatin”, “simvastatin”, “pravastatin”, “lovastatin”, “fluvastatin”, “hydroxymethylglutaryl-CoA”, “percutaneous coronary intervention”, “PCI”, “stent” and “randomized”. We also reviewed the references within related meta-analyses. The primary endpoints of the analysis were the incidences of PMI and MACE. The definition of PMI was taken from the original articles. MACE included death, spontaneous myocardial infarction (MI), and target vessel revascularization (TVR) at the longest follow-up for each study. Studies were included in the present meta-analysis if they met the following criteria: (1) the studies were written in English; (2) the studies were RCTs conducted on humans; (3) the studies did not include patients undergoing primary PCI and non-ST elevation acute coronary syndrome (NSTE-ACS) with high-risk features needing emergency coronary angiography; (4) the studies included a high-dose statin pretreatment therapy group and a no statin or low-dose statin pretreatment therapy group; (5) the definition of PMI in the studies was specified; (6) the incidence of PMI or MACE was specified or could be calculated.

### Data extraction and quality assessment

The data extraction was independently performed by WL and PPA. Any disagreement was resolved by consensus. The risk of bias in each study was evaluated by using the Cochrane Collaboration's instructions [20]. Quality assessment was made on allocation concealment, similarity of baseline characteristics, eligibility criteria, blinding, completeness of follow-up and intention-to-treat analysis. Study quality was quantified using the Jadad score [21].

### Statistical analysis

The statistical analyses were performed using Review Manager 5.2.0. To evaluate the clinical benefit of high-dose statin specifically for patients undergoing elective PCI, subgroup analyses were performed on trials that included only patients with stable angina or patients with ACS, only statin-naive patients, or only patients with prior low-dose statin treatment. The incidence of PMI and MACE was expressed in dichotomous variables, and the results were expressed as odds ratios (OR) with 95% confidence intervals (CI). Heterogeneity across trials was evaluated with I^2^ statistic, defined as I^2^>50%. If heterogeneity existed a random-effect model was used; otherwise, a fixed-effect model was chosen. Potential publication bias was assessed with funnel plots. All tests were two-tailed, and a P value of less than 0.05 was regarded as statistically significant.

## Results

### Characteristics of studies selected for meta-analysis

**Figure 1:**
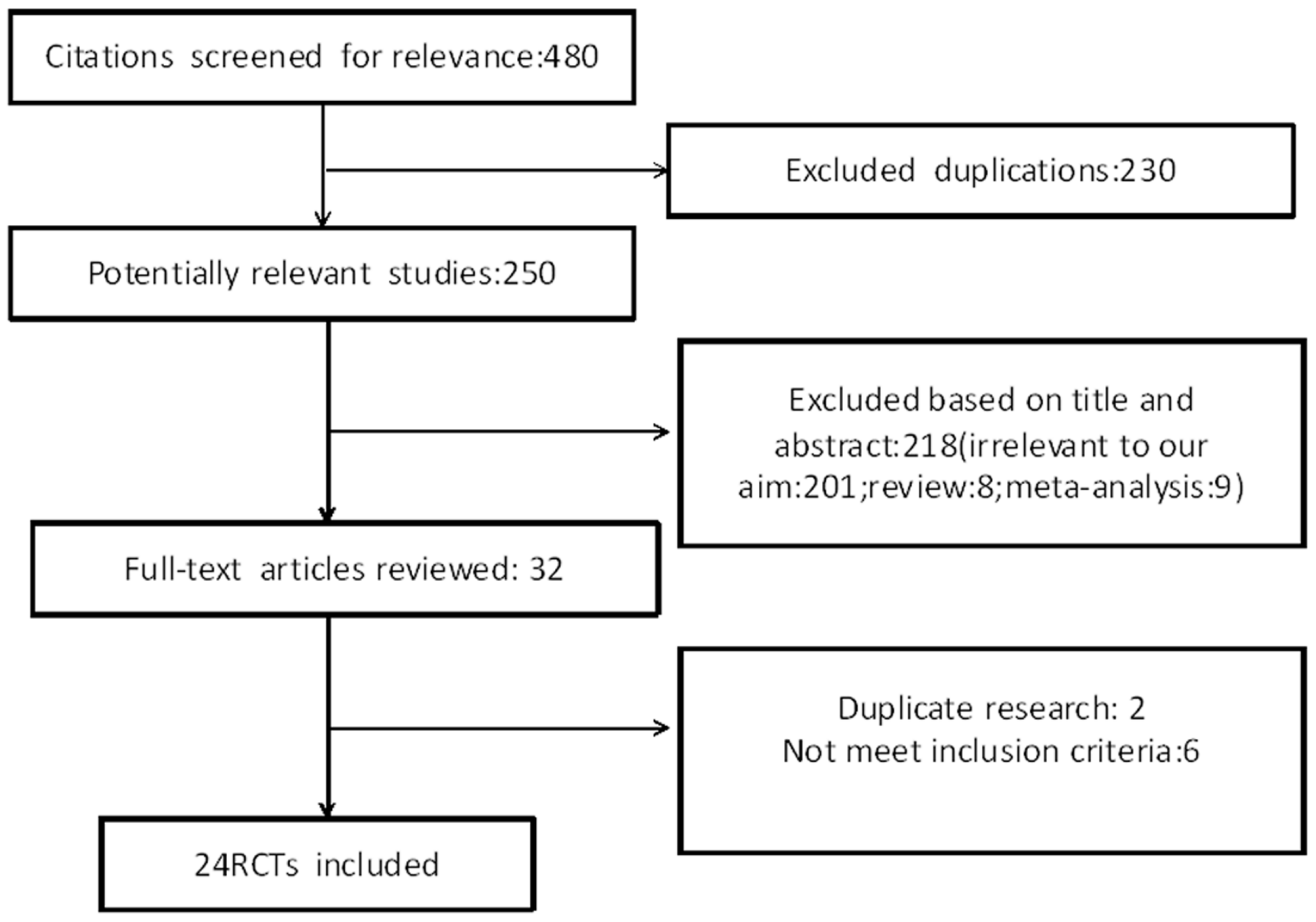
Study selection diagram. 480 potentially relevant publications were identified from Pubmed/Medline search for key words and research references within related studies. Of these, 230 were duplications and 218 were excluded after further examination of the title and abstract. Of the 32 remaining articles, 2 were from duplicate RCTs and 6 did not meet our inclusion criteria, which left 24 RCTs that were used for our analysis.

We identified 480 potentially relevant articles from the PubMed/Medline database (Figure 1). After reading the titles and abstracts of all potential articles, the full text of 32 articles was reviewed. By further evaluation, two articles were excluded as duplicate research; and six additional articles were excluded for not meeting our inclusion criteria. Finally, a total of 24 RCTs were examined.

Tables 1, 2 and 3 summarize the main characteristics of the 24 RCTs included in this study. All included studies were published between 2004 and 2013. 5,526 patients were included in the analysis: 2,867 were randomized to a high-dose statin group and 2,659 randomized to receive low-dose or no statin therapy (Control group). Approximately 73% of enrolled patients were male, 30% had diabetes, roughly two-thirds of the patients had chronic stable angina, and one-third of the patients presented with ACS. There were five trials [18], [22]–[25] with only prior low dose statin-treated patients, eighteen [16], [17], [26]–[41] with only statin-naïve patients, and one [42] with both prior low-dose statin-treated and statin-naïve patients. Twelve studies [16], [18], [22], [24], [26], [29], [30], [33], [34], [36], [39], [42] included patients with stable angina, seven studies [17], [28], [35], [37], [38], [40], [41] included patients with ACS, and five studies [23], [25], [27], [31], [32] included patients with mixed presentations. Fourteen trials [16]–[18], [22]–[28], [30], [31], [34], [37] used atorvastatin, eight [24], [33], [35], [38]–[42] used rosuvastatin, three [27], [29], [36] used pravastatin, one [27] used fluvastatin, and two [27], [32] used simvastatin. The duration of statin pretreatment ranged from two hours to four weeks. All patients in these trials received statin therapy after PCI, irrespective of the initial assignment. Eighteen studies [17], [18], [22], [23], [25]–[29], [31]–[34], [36], [37], [40]–[42] included short-term follow-up (in-hospital or up to 30 days), and six studies [16], [24], [30], [35], [38], [39] had follow-up duration between six months and forty-five months. The definition of PMI varied among the included studies as follows: Four studies [26], [28], [35], [37] demanded a CK-MB concentration greater than two times above the upper normal limit (ULN), thirteen studies [17], [18], [23], [24], [29]–[31], [33], [34], [38], [39], [41], [42] a CK-MB concentration greater than three times above ULN, and one study [27] a CK-MB concentration greater than five times above ULN. Other studies employed the Troponin I (TnI) concentration to define PMI. Three studies [16], [32], [40] required a TnI concentration greater than three times above ULN and two studies [25], [26] a TnI concentration greater than five times above ULN. Table 4 shows the quality characteristics of the included RCTs. Seventy-nine percent of the trials are high-quality trials, according to the Jadad score. Three trials [23], [25], [28] used allocation concealment and blinding.

**Table 1:** Characteristics of included studies.

Trial	patient (n)	Type of population	Clinical presentation	Type of statin	Statin regime before PCI	Statin regime after PCI	Follow up	PMI definition
armyda	153	Statin naive	Stable angina	Atorvastatin	7-day 40 mg/d before PCI VS placebo	Atorvastatin 40 mg/d	30days	CK-MB>2UNL
Briguori et al.	451	Statin naive	92% Stable angina/asymptomatic; 8% unstable angina	Atorvastatin29%;pravastatin,29%, simvastatin,39%, fluvstatin, 3%	3-Day pretreatment(average 17 days) VS no statin pretreatment	Same statin as before PCI in the statin group and atorvastatin 20 mg/d in the control group	In-hospital	CK-MB>5UNL
armyda-acs	171	Statin naive	NSTE-ACS	Atorvastatin	80 mg 12 h before PCI+40 mg 2 h before PCI VS placebo before PCI	Atorvastatin 40 mg/day	30days	CK-MB>2UNL
Bozbas et al.	93	Statin naive	Stable angina	Pravastatin	1-week pretreatment with 10 mg/d VS 40 mg/d before PCI VS no statin pretreatment	Pravastatin 10 to 40 mg/d	In-hospital	CK-MB>3UNL
Kinoshita et al.	42	Statin naive	Stable angina	Atorvastatin	5–20 mg/d ≥2 wk before PCI to reach LDL<70 mg/dl VS 100 mg/dl	Atorvastatin 5 to 20 mg/d	6months	CK-MB>3UNL
NAPLES II	668	Statin naive	98% Stable angina/asymptomatic;2% unstable angina	Atorvastatin	80 mg within 24 h before PCI VS no statin pretreatment	atorvastatin 20 mg/day	In-hospital	CK-MB>3UNL
armyda-recapture	383	Statin-treated	53% stable angina and 47% NSTE-ACS	Atorvastatin	80 mg 12 h before PCI+40-mg 2 h before PCI VS placebo	Atorvastatin 40 mg/day	30days	CK-MB>3UNL
Jia et al.	228	Statin naive	70.6% NSTE-ACS;29.4%STE-ACS	Simvastatin	7-day pretreatment with 80 mg/d before PCI VS 20 mg/d before PCI	Simvastatin 20 mg/d	In-hospital	TnI>3UNL
Cay et al.	299	Statin naive	Stable angina	Rosuvastatin	Rosuvastatin 40 mg 24 h before PCI VS no statin pretreatment	Rosuvastatin 10–40 mg/d	In-hospital	CPK-MB>3UNL
Toso et al.	161	Statin naive	Stable angina	Atorvastatin	80 mg within 48 h before PCI VS placebo within 48 h before PCI	NA	In-hospital	CPK-MB>3UNL
Yun et al.	445	Statin naive	NSTE-ACS	Rosuvastatin	40 mg within 7–25 h before PCI VS no statin treatment before PCI	Rosuvastatin 10 mg/day	12months	CK-MB>2UNL
Veselka et al.	200	Statin naive	Stable angina	Atorvastatin	2-day pretreatment with atorvastatin 80 mg/day before PCI VS without atorvastatin pretreatment	atorvastatin 20 to 80 mg/day	45months	TnI>3UNL

PCI = percutaneous coronary intervention; CK-MB = creatine kinase-MB; UNL = upper normal limit of normal; NSTE-ACS = non-ST-segment elevation acute coronary syndrome;

**Table 2:** Characteristics of included studies.

Trial	Patient (n)	Type of population	Clinical presentation	Type of statin	Statin regime before PCI	Statin regime after PCI	Follow up	PMI definition
Fujii et al.	80	Statin naive	Stable angina	Pravastatin	4-week pretreatment with 20 mg/d before PCI VS no pretreatment	NA	In-hospital	TnI>5UNL
Yu et al.	81	Statin naive	NSTE-ACS	Atorvastatin	80 mg 12 h before PCI+40 mg before PCI VS placebo group	Atorvastatin 20 mg/d	30days	CK-MB>2UNL
Gao et al.	117	Statin naive	NSTE-ACS	Rosuvastatin	20 mg 12 h before PCI +10 mg 2 h before PCI VS no treatment	Rosuvastatin 10 mg/d	6months	CK-MB>3UNL
Jang et al.	335	Statin naive	NSTE-ACS	Atorvastatin	80 mg 12 h and 40 mg 2 h before PCI VS no statin pretreatment	Atorvastatin 40 mg/day	30days	CK-MB>3UNL
Zemánek et al.	202	Statin-treated	Stable angina	Atorvastatin	7-day pre-treatment with 80 mg/d VS no statin pretreatment	NA	In-hospital	CK-MB>3UNL
ROMA Trial	160	Statin naive	Stable angina	Rosuvastatin	Rosuvastatin 40 mg within 24 h before PCI VS no statin	Rosuvastatin 20 mg/d	12months	CK-MB>3UNL
ROMA II trial	450	Statin-treated	Stable angina	Atorvastatin; Rosuvastatin	Rosuvastatin 40 mg or Atorvastatin 80 mg 24 h before PCI VS no statin pretreatment	Rosuvastatin 20 mg/d Atorvastatin 40 mg/d	12months	CK-MB>3UNL
Nafasi et al.	190	Statin-treated	Stable angina and recent MI	Atorvastatin	80 mg within 24 h before PCI VS placebo within 24 h before PCI	NA	In-hospital	TnI>5UNL
Takano et al.	210	statin-treated and statin-naive	Stable angina	Rosuvastatin	5–7day pretreatment with 20 mg/d before PCI VS 5–7day pretreatment with 2.50 mg before PCI	Rosuvastatin 10 mg/d AND rosuvastatin 2.5 mg/d	In-hospital	CK-MB>3UNL
Wang et al.	125	Statin naive	NSTE-ACS	Rosuvastatin	20 mg 2–4 hours before PCI VS placebo before PCI	Rosuvastatin 10 mg/d	30days	CK-MB>3UNL
Luo et al.	67	Statin naive	NSTE-ACS	Rosuvastatin	20 mg 12 h before PCI+20 mg 2 h before PCI VS no Statin pretreatment	Rosuvastatin 10 mg/d	30days	TnI>3UNL
Li et al.	215	statin-treated	Stable angina	Atorvastatin	80 mg 12 h before PCI VS 20 mg 12 h before PCI	Atorvastatin 20 mg/d	30days	NA

PCI = percutaneous coronary intervention; CK-MB = creatine kinase-MB; UNL = upper normal limit of normal; NSTE-ACS = non-ST-segment elevation acute coronary syndrome; ROMA trial = Rosuvastatin pretreatment in patients undergoing elective PCI to reduce the incidence of periprocedural myocardial necrosis; ROMA II trial = Comparison of high reloading Rosuvastatin and Atorvastatin pretreatment in patients undergoing elective PCI to reduce the incidence of periprocedural myocardial necrosis; NA = not available.

**Table 3:** Clinical and Procedural features in the overall population.

Variables	High-dose statin	Control
Population size	2867	2659
Men	2103(73)	1921(72)
Family history of CAD	536(19)	405(15)
Hypertension	1935(67)	1736(65)
Diabetes Mellitus	868(30)	766(29)
Hyperlipidemia	701(24)	531(20)
Smoking	772(27)	689(26)
Previous myocardial infarction	440(15)	455(17)
Previous PCI	219(8)	194(7)
Previous CABG	94(3)	93(3)
Clinical presentation		
Chronic stable angina	1950(68)	1739(66)
NSTEACS	886(31)	884(33)
STEACS	31(1)	36(1)
Medical therapy		
β-blocker	1318(46)	1318(50)
ACEI/ARB	1193(42)	1196(45)
CCB	331(12)	326(12)
Vessel disease		
Multiple vessel	727(25)	688(26)
B2/C lesion	1289(45)	1088(41)
Thrombus	40(1.4)	29(1.1)
Treated vessel		
LM	14(0.5)	17(0.7)
LAD	1250(47)	1178(49)
LCX	598(23)	532(22)
RCA	778(29)	663(28)
Sapheonus vein graft	6(0.2)	11(0.5)
Multiple vessel PCI	280(10)	268(10)
Antithrombotic therapy		
Aspirin	2674(93)	2416(91)
Clopidogrel	2460(86)	2188(82)
Glycoprotein IIb/IIIa inhibitors	295(10)	315(12)

CAD = coronary artery disease; PCI = percutaneous coronary syndrome; CABG = coronary artery bypass graft; NSTEACS = non-ST segment elevation acute coronary syndrome; STEACS = ST segment elevation acute coronary syndrome; ACEI = angiotensin converting enzyme inhibitor; ARB = Angiotensin II receptor antagonist; CCB = calcium channel blocker; LM = left main; LAD = left anterior descending; LCX = left circumflex; RCA = right coronary artery.

**Table 4:** Quality of included RCTs.

Author, Year	Jadad score	Allocation Concealment	Similarity of baseline characteristics	Eligibility Criteria	Blinding			Completeness of Follow-up	Intention-to-treat
					Outcome Assessor	Care provider	Patient		
ARMYDA,2004	5	NA	Yes	Yes	No	Yes	Yes	Yes	Yes
Briguori,2004	4	Yes	Yes	Yes	No	No	No	Yes	Yes
ARMYDA-ACS 2007	6	Yes	Yes	Yes	No	Yes	Yes	Yes	Yes
Kinoshita,2007	4	NA	Yes	Yes	No	No	NA	Yes	Yes
Bozbas,2007	4	NA	Yes	Yes	No	No	Yes	Yes	Yes
Naples II,2009	5	Yes	Yes	Yes	No	No	No	Yes	Yes
ARMYDA-RECAPTURE,2009	6	Yes	Yes	Yes	No	Yes	Yes	Yes	Yes
Jia,2009	4	NA	Yes	Yes	Yes	No	No	Yes	Yes
Cay,2010	3	NA	Yes	Yes	No	No	No	Yes	Yes
Toso,2011	3	No	Yes	Yes	No	No	No	Yes	Yes
Yun,2011	4	NA	Yes	Yes	No	No	No	Yes	Yes
Veselka,2011	3	NA	Yes	Yes	No	No	No	Yes	Yes
Fujii,2011	4	NA	Yes	Yes	No	No	No	Yes	Yes
Yu,2011	4	NA	Yes	Yes	No	Yes	Yes	Yes	Yes
Gao,2012	3	NA	Yes	Yes	No	No	No	Yes	Yes
Jang,2013	4	NA	Yes	Yes	No	No	Yes	Yes	Yes
Zemánek,2013	4	NA	Yes	Yes	No	No	Yes	Yes	Yes
ROMA II,2013	5	Yes	Yes	Yes	No	No	No	Yes	Yes
Nafasi,2013	6	Yes	Yes	Yes	Yes	Yes	Yes	Yes	Yes
ROMA,2013	4	Yes	Yes	Yes	No	No	No	Yes	Yes
Takano,2013	3	No	Yes	Yes	No	No	No	Yes	Yes
Wang,2013	4	NA	Yes	Yes	Yes	Yes	Yes	Yes	Yes
Luo,2013	4	NA	Yes	Yes	NA	NA	NA	Yes	Yes
Li,2013	4	NA	Yes	Yes	NA	NA	NA	Yes	Yes

NA = not available.

### Effect of statin pretreatment on clinical outcome

**Figure 2:**
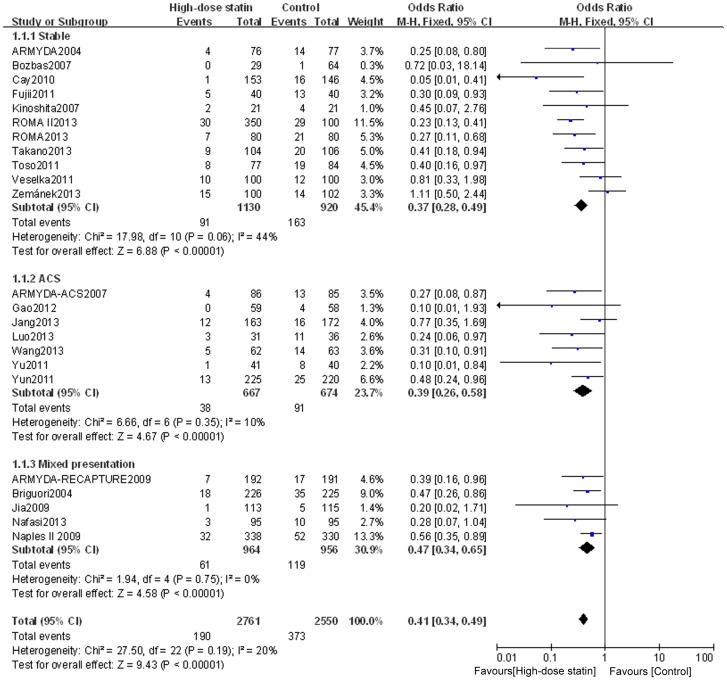
Forest plots for PMI incidence for patients stratified by different clinical presentation. Forest plots were generated using Review Manager 5.2.0 for patients with stable angina, ACS or mixed disease presentation from individual and pooled trials. The incidence of PMI was expressed as a dichotomous variable, and the results were expressed as odds ratios (OR) with 95% confidence intervals (CI). Heterogeneity across trials was evaluated with I^2^ statistic, defined as I^2^>50%. Because the heterogeneity was low, a fixed-effect model was used.

**Figure 3:**
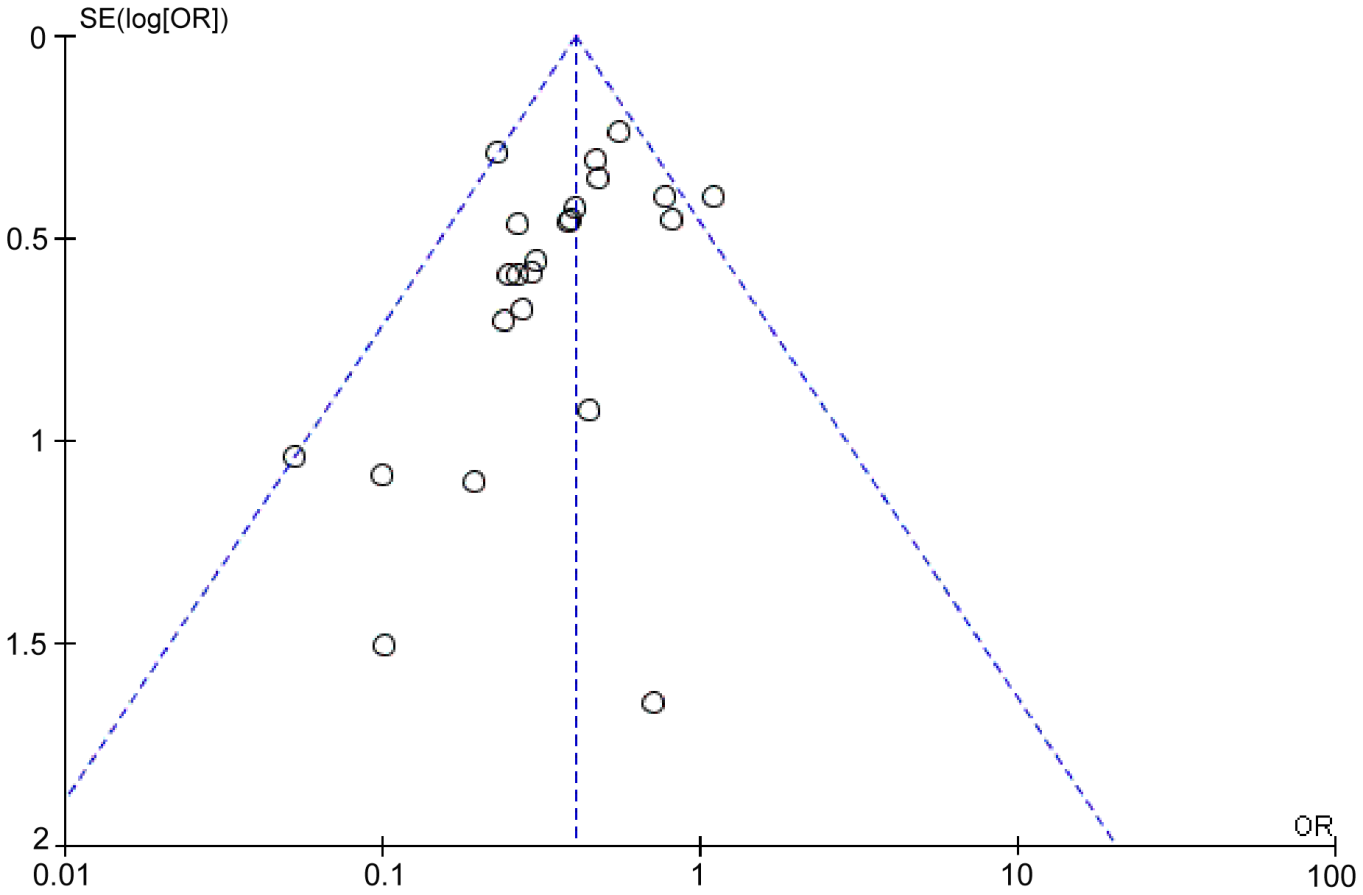
Funnel plot for PMI incidence to rule out publication bias. Funnel plot was generated using a fixed-effect model by Review Manager 5.2.0.

Outcome data for PMI were available from 23 RCTs, but not from the study by Li Q et al. [22]. The overall outcomes, based on the fixed effects model, showed that high-dose statin pretreatment was associated with a 59% relative reduction in PMI (OR = 0.41, 95% CI 0.34–0.49, P<0.00001; I^2^ = 20%) (Figure 2). The relative symmetry in the funnel plot shows that there was no evidence to suggest any publication bias (Figure 3).

**Figure 4:**
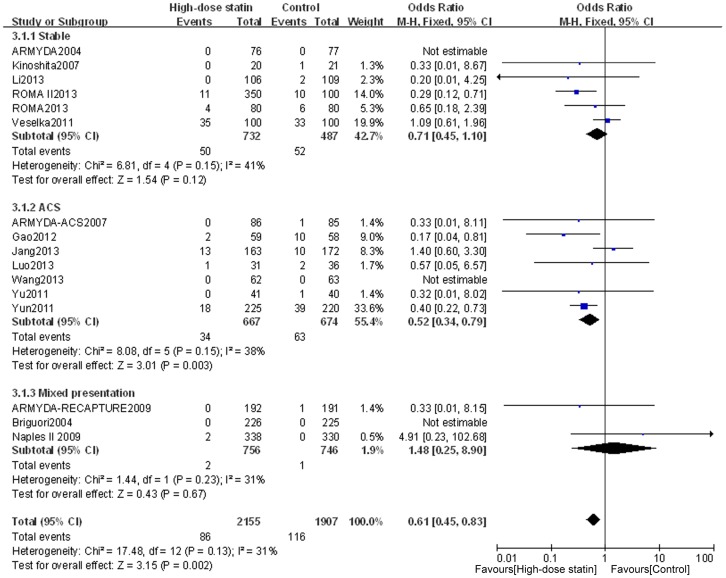
Forest plots for MACE incidence for patients stratified by different clinical presentation. Forest plots were generated using Review Manager 5.2.0 for patients with stable angina, ACS or mixed disease presentation from individual and pooled trials. The incidence of MACE was expressed as a dichotomous variable, and the results were expressed as odds ratios (OR) with 95% confidence intervals (CI). Heterogeneity across trials was evaluated with I^2^ statistic, defined as I^2^>50%. Because the heterogeneity was low, a fixed-effect model was used.

**Figure 5:**
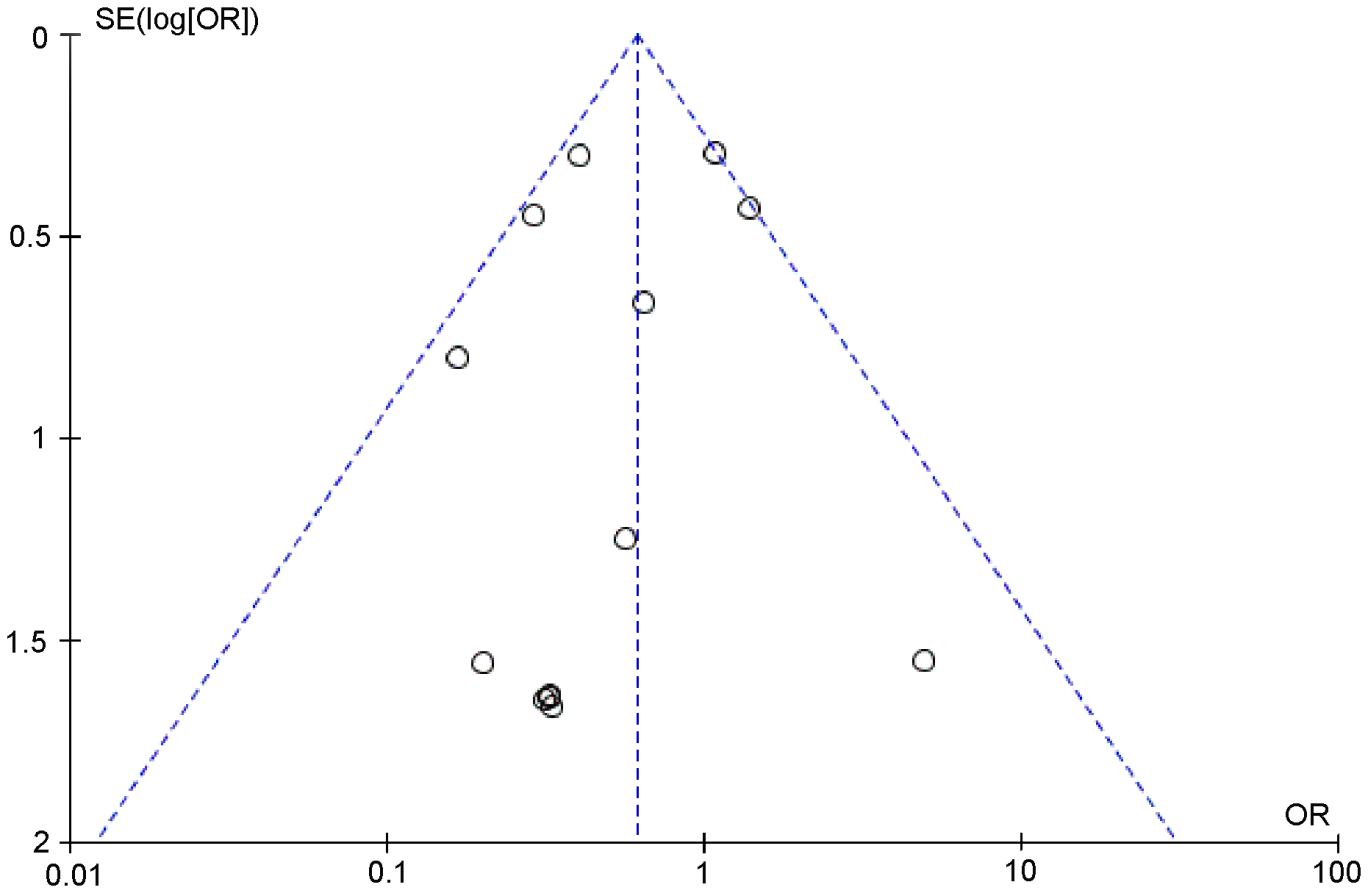
Funnel plot for MACE incidence to rule out publication bias. Funnel plot was generated using a fixed-effect model by Review Manager 5.2.0.

Outcome data for MACE were available from 16 RCTs, whereas the other 8 RCTs (by Bozbas et al. [29], Jia et al. [32], Cay et al. [33], Toso et al. [34], Fujii et al. [36], Zemanek et al. [18], Nafasi et al. [25], and Takano et al. [42]) only collected data about PMI. The overall outcomes, based on the fixed effects model, showed that high-dose statin pretreatment was associated with a 39% relative reduction in MACE (OR = 0.61, 95% CI 0.45–0.83, P = 0.002; I^2^ = 31%) (Figure 4). There was no evidence of significant heterogeneity among the 16 trials (P = 0.13; I^2^ = 31%), and the funnel plot analysis did not suggest the presence of any publication bias (Figure 5). These data confirm the reliability of this set of data and also verify the overall beneficial effects of high-dose statin loading on PMI and MACE using a greatly expanded data set relative to previous meta-analyses [14], [19].

### Analysis of subgroups with different types of disease presentation

To determine whether the outcome of high-dose statin pretreatment prior to PCI differs for patients with stable angina, ACS, or a mixed disease presentation, we assessed subgroups of patients according to their disease classification. High-dose statin pretreatment was associated with 63% relative reduction in PMI for patients with stable angina (OR = 0.37, 95% CI 0.28–0.49, P<0.00001; I^2^ = 44%); 61% relative reduction in PMI for patients with ACS (OR = 0.39, 95% CI 0.26–0.59, P<0.00001; I^2^ = 10%); and 53% relative reduction in PMI for patients with mixed presentation (OR = 0.47, 95% CI 0.34–0.65, P<0.00001; I^2^ = 0%) (Figure 2).

We also assessed the stable angina, ACS, and mixed disease subgroups for their effect on MACE. For the subgroup with ACS, the incidence of MACE was significantly lower in the high-dose statin group than in the control group (OR = 0.52, 95% CI 0.34–0.79, P = 0.003; I^2^ = 38%); however, for patients with stable angina or mixed presentation, there was no significant difference in MACE between the high-dose statin group and the control group (OR = 0.71, 95% CI 0.45–1.10, P = 0.12; I^2^ = 41%; and OR = 1.48, 95% CI 0.25–8.90, P = 0.67; I^2^ = 31%, respectively) (Figure 4). These findings confirm and extend previous findings that suggest that high dose statin loading is only effective for ACS patients on MACE [19].

### Analysis of subgroups according to prior low-dose statin use

**Figure 6:**
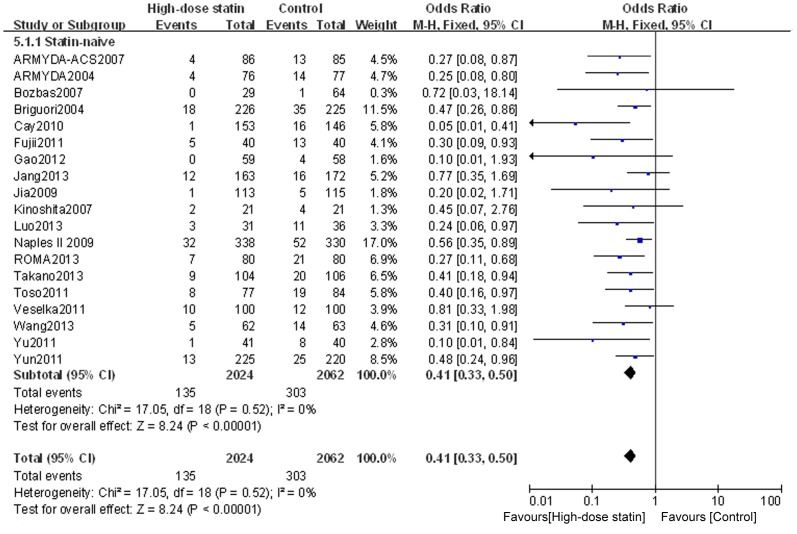
Funnel plot for PMI incidence for statin naïve patients. Forest plot was generated using Review Manager 5.2.0 for statin naïve patients from individual and pooled trials. The incidence of PMI was expressed as a dichotomous variable, and the results were expressed as odds ratios (OR) with 95% confidence intervals (CI). Heterogeneity across trials was evaluated with I^2^ statistic, defined as I^2^>50%. Because the heterogeneity was low, a fixed-effect model was used.

**Figure 7:**
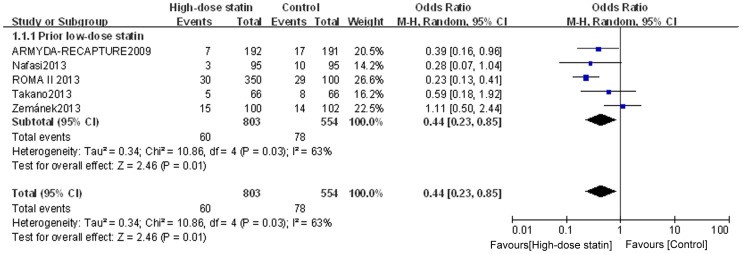
Funnel plot for PMI incidence for patients with prior low-dose statin use. Forest plot was generated using Review Manager 5.2.0 for patients with prior low-dose statin use from individual and pooled trials. The incidence of PMI was expressed as a dichotomous variable, and the results were expressed as odds ratios (OR) with 95% confidence intervals (CI). Heterogeneity across trials was evaluated with I^2^ statistic, defined as I^2^>50%. A random-effect model was used for the subgroup with prior low-dose statin treatment because heterogeneity existed for this group.

**Figure 8:**
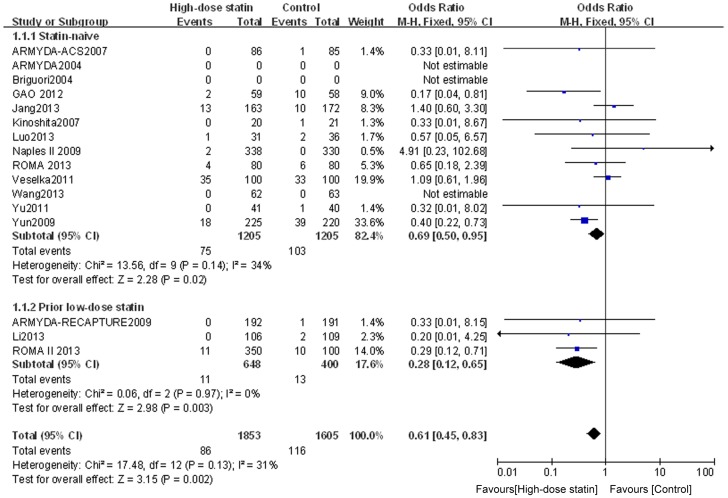
Forest plots for MACE incidence for patients stratified by prior low-dose statin use. Forest plots were generated using Review Manager 5.2.0 for statin naïve patients or patients with prior low-dose statin use from individual and pooled trials. The incidence of MACE was expressed as a dichotomous variable, and the results were expressed as odds ratios (OR) with 95% confidence intervals (CI). Heterogeneity across trials was evaluated with I^2^ statistic, defined as I^2^>50%. Because the heterogeneity was low, a fixed-effect model was used.

To determine whether chronic low-dose statin therapy prior to the high-dose PCI pretreatment affects clinical outcome, we grouped the patients according to whether or not they had a history of low-dose statin treatment prior to the high-dose pre-PCI treatment. For the subgroup of statin-naïve patients, high-dose statin pretreatment was associated with a lower incidence of PMI (OR = 0.41, 95% CI 0.33–0.50, P<0.00001; I^2^ = 0%) (Figure 6). For the subgroup with prior low-dose statin treatment, heterogeneity was found across trials (I^2^ = 63%), and a randomized model was therefore used. Similar to the results for the statin-naïve patients, the incidence of PMI for the prior low-dose statin patients was lower in the high-dose statin group than that in the control group (OR = 0.44, 95% CI 0.23–0.85, P = 0.01) (Figure 7). Furthermore, assessment of the effects on MACE suggested that high-dose statin loading is beneficial for both statin-naïve patients (OR = 0.69, 95% CI 0.50–0.95, P = 0.02; I^2^ = 34%) and prior low-dose statin patients (OR = 0.28, 95% CI 0.12–0.65, P = 0.003; I^2^ =  0%) (Figure 8). These data provide evidence using meta-analysis or multiple RCTs to support the use of high-dose statin loading prior to PCI for patients having prior low-statin chronic therapeutic use.

### Analysis of the effects of high-dose statins on follow-up clinical events for subgroups of patients

To determine the effects of high-dose statin loading on follow-up clinical events, we assessed the number of patients undergoing death, spontaneous MI, TVR or MACE events in the follow-up period after PCI. High-dose statin treatment did not significantly reduce overall mortality or spontaneous MI (P = 0.15 and P = 0.37, respectively), with similar findings for subgroups stratified according to disease type or prior statin use (Table 5). TVR was significantly reduced by high-dose statin pretreatment (overall P = 0.02), with high-dose statin pretreatment most effective for the ACS group and the prior low-dose statin group (P = 0.01 and P = 0.04, respectively). A statistical difference in the incidence of MACE was also observed overall (P = 0.002), for both statin-naïve (P = 0.02) and prior low-dose statin (P = 0.003) patients, and for patients with ACS (P = 0.003), but not for patients with stable angina (P = 0.12). These results provide additional support for the applicability of high-dose statins for patients with ACS, for both statin-naïve and prior low-dose statin patients.

**Table 5:** Clinical events in follow-up.

Events	High-dose statin n (%)	Control n (%)	P
Stable			
Death	12(1.6)	13(2.7)	0.67
Spontaneous MI	7(1.0)	7(1.3)	0.50
TVR	31(4.2)	32(6.6)	0.23
MACE	50(6.8)	52(10.7)	0.12
ACS			
Death	3(0.4)	9(1.3)	0.12
Spontaneous MI	15(2.2)	20(3.0)	0.40
TVR	16(2.4)	34(5.0)	0.01
MACE	34(5.0)	63(9.3)	0.003
Mixed presentation			
Death	1(0.1)	1(0.1)	0.50
Spontaneous MI	1(0.1)	0(0)	0.51
TVR	0(0)	0(0)	NA
MACE	2(0.2)	1(0.1)	0.67
Statin-naive			
Death	13(1.1)	20(1.6)	0.28
Spontaneous MI	21(1.7)	24(2.0)	0.80
TVR	41(3.4)	59(4.9)	0.09
MACE	75(6.2)	103(8.5)	0.02
Prior low-dose statin			
Death	3(0.5)	3(0.8)	0.25
Spontaneous MI	2(0.3)	3(0.8)	0.07
TVR	6(0.9)	7(1.7)	0.04
MACE	11(1.7)	13(3.3)	0.003
Overall			
Death	16(0.7)	23(1.2)	0.15
Spontaneous MI	23(1.1)	27(1.4)	0.37
TVR	47(2.2)	66(3.5)	0.02
MACE	86(4.0)	116(6.1)	0.002

Spontaneous MI = spontaneous myocardial infarction; ACS = acute coronary syndrome;

TVR = target vessel revascularization; MACE = major adverse cardiovascular events;

NA = not available.

## Discussion

This meta-analysis indicated that high-dose statin pretreatment can result in a significant reduction of PMI and cardiovascular events in patients undergoing elective PCI. This basic finding is consistent with previous meta-analysis studies [14], [19]; however, our study includes more RCTs and more than 1200 additional patients and thus provides increased statistical power. When stratified according to clinical presentation, the positive effect was significant for patients with ACS, as previously suggested [19]. However, to our knowledge, our study is unique in that we provide the first meta-analysis showing that the positive effect of high-dose statin pretreatment on PMI and MACE (excluding PMI) is significant for both statin-naive patients and patients with prior low-dose statin therapy.

Previous meta-analyses show that the elevation of cardiac markers after PCI correlates with an increase of clinical events during follow-up [4], [43]. There is still no consensus on the definition of PMI; however, we have elected to analyze all relevant studies with quality data to provide the most comprehensive analysis possible. Despite variability in the definition of PMI, this meta-analysis demonstrated that high-dose statin pretreatment before PCI reduces the incidence of PMI. The positive effect of high-dose statin pretreatment on PMI was consistent in patients with stable and unstable coronary artery disease as well as in statin-naïve patients and prior statin-treated patients. In addition, high-dose statin pretreatment was associated with a 39% relative reduction in clinical events. The benefit in clinical events was predominantly driven by the reduction in TVR, especially for patients with ACS. The incidence of death and spontaneous MI was lower in the high-dose statin group. High-dose statin loading prior to PCI did not reduce the clinical events in patients with stable coronary artery disease. Our study confirms that reloading high-dose statin improves the clinical outcome in patients undergoing long-term therapy with statins.

Although the exact mechanisms underlying the early protective effects of high dose stains in cardiovascular events remain undetermined, the benefit of statins can be attributed to its pleiotropic effects, which include anti-inflammation, anti-platelet aggregation, improvement of endothelial function, and plaque stability. Periprocedural inflammatory status predicts early and late adverse outcomes after PCI [44]–[47]. Studies showed that a reduction of periprocedural myocardial injury after high-dose pretreatment is associated with attenuated inflammatory response [40], [48], [49]. This may explain why patients with ACS may benefit most from high-dose statins therapy before PCI. A clinical study [50] found that long-term treatment with statins did not result in a reduction of PMI in patients undergoing elective PCI. Animal experiments showed that the cardioprotection of statins disappears with time and that high-dose statin reloading before ischemia/reperfusion can restore the waning protection [51]. This may suggest that high-dose statin pretreatment is needed to reach the desired pleiotropic effects for patients under long-term low-dose statin treatment.

The main conclusion of this meta-analysis was similar with that from Patti et al. [14] and Alexandre et al. [19]. However, these other results should be interpreted with caution. Compared with the meta-analysis by Patti et al., we excluded the STATIN STEMI trial [52]. To our knowledge, it is the only study to evaluate the impact of high-dose statin pretreatment before primary PCI in patients with STEMI. The trial could not provide sufficient power to determine a significant difference in PMI between high-dose and low-dose statin. In their meta-analysis, Patti et al. demonstrated that high-dose statin loading before PCI significantly reduced PMI and MACE within thirty days. The clinical benefit was mainly driven by the reduction in PMI. However, when PMI was excluded from the end points, there was no significant difference in the rate of MACE within thirty days between high-dose statin and control groups (OR = 0.44, P = 0.05). Our study demonstrates that high-dose statin pretreatment before PCI significantly reduces clinical events, including spontaneous MI, death, and TVR. Furthermore, we showed that high-dose statin reloading before PCI significantly reduces the occurrence of PMI and MACE during follow-up in prior low-dose statin-treated patients. Unlike the meta-analysis by Alexandre et al. [19], our study included patients under long-term low-dose statin therapy, RCTs that compared high-dose with low-dose statin treatment, and RCTs that compared different types of statin. In clinical practice, patients under long-term statin therapy constitute a large proportion of patients who undergo elective PCI. Moreover, our study included recent large scale trials [17], [24]. Therefore, our study confirms and extends the conclusion of the meta-analysis by Alexandre et al.

The results of our meta-analysis are not consistent with the conclusions of Veselka et al. [15], [16], Jang et al. [17] and Zemanek et al. [18], which showed that high-dose statin pretreatment before PCI did not reduce the incidence of PMI and MACE in patients undergoing PCI. We think that this disagreement can be explained by different doses and durations of statins. As the optimal doses and duration of statins have not been determined, different therapeutic application may result in different results. Another potential explanation for the controversial outcome of the studies by Veselka et al. and Zemanek et al. is that the cardiac marker was examined at a later point in time (16 to 24 hours after PCI), potentially missing the opportunity to identify PMI. In addition, the discordant results may be attributed to a lower incidence of increased CK-MB in the high-dose statin loading group, compared to the control group.

There are several limitations in this meta-analysis. First, the studies used different definitions of PMI and different treatment strategies. To date, it is not possible to find an ideal marker for a universally accepted definition of PMI. Different doses, durations and types of statins may have different effects on PMI and MACE. A standardized statin protocol would help to eliminate the profounder. Secondly, although many studies provided data about PMI and MACE, one out of the twenty-four studies did not report the incidence of PMI, and eight out of twenty-four studies did not provide the incidence of MACE. Finally, we did not have access to patient-level data; therefore, we did not exclude the influence of different antithrombotic strategies.

## Conclusions

High-dose statin pretreatment can result in a significant reduction in PMI and cardiovascular events in patients undergoing elective PCI. The positive effects of high-dose statin pretreatment on PMI and MACE are significant for statin-naive and prior low-dose statin-treated patients. The positive effect of high-dose statin pretreatment on MACE is significant for patients with ACS. The information provided in this analysis should be useful in the selection of patients who have the greatest potential for success in the use of high-dose statin loading prior to PCI. In particular, we suggest that high-dose pretreatment should be used regardless of prior low-dose statin use for patients with ACS, but that the high-dose statin loading can be eliminated for patients with stable angina to avoid unnecessary statin exposure and unwarranted expenditure on statins.

## Supporting Information

Checklist S1 PRISMA Checklist.
(DOC)
